# Isolation and Characterization of *Rickettsia finnyi*, Novel Pathogenic Spotted Fever Group *Rickettsia* in Dogs, United States

**DOI:** 10.3201/eid3111.250681

**Published:** 2025-11

**Authors:** Praveen K. Korla, Michael G. Karounos, Sarah B. Clarke, Cynthia Robveille, James M. Wilson, Edward B. Breitschwerdt, Adam J. Birkenheuer, Barbara A. Qurollo

**Affiliations:** North Carolina State University College of Veterinary Medicine, Raleigh, North Carolina, USA

**Keywords:** spotted fever group *Rickettsia*, bacteria, zoonoses, culture, genome, rickettsiosis, tickborne disease, vector-borne infections, United States

## Abstract

In 2020, a novel spotted fever group *Rickettsia* was described in 3 clinically ill dogs in the United States. Using naturally infected canine blood, the novel *Rickettsia* sp. was isolated in epithelial (Vero E6) and mononuclear (DH82 and 030D) cell lines. The sequenced whole genome revealed a 1.27 Mb circular chromosome with 96.87% identity to *Rickettsia raoultii* on the basis of average nucleotide identity analysis. A maximum-likelihood phylogeny tree placed the novel *Rickettsia* in its own branch within the spotted fever group. Immunofluorescence revealed single rods localized along the membrane in epithelial cells and randomly distributed in the cytoplasm of mononuclear cells. We propose the name *Rickettsia finnyi* sp. nov., strain 2024-CO-Wats, which is available from national and international Rickettsial isolate reference collections. Fever and thrombocytopenia were among abnormalities in the 17 naturally infected dogs we describe, underscoring the pathogenic importance of *R. finnyi* sp. nov. and its potential public health relevance.

In 2020, a unique spotted fever group *Rickettsia* (SFGR), *Rickettsia* sp. 2019-CO-FNY, was identified in 3 clinically ill dogs in the southern and midwestern United States (*1*). Those dogs exhibited symptoms like those caused by *R. rickettsii*, the agent responsible for Rocky Mountain spotted fever (RMSF). SFGR are emerging tickborne pathogens infecting dogs and humans. Among tickborne pathogens infecting dogs, SFGR had the highest seroprevalence at 10.4% in the United States during 2004–2010 (*2*). The Centers for Disease Control and Prevention reported annual SFGR cases in humans in the United States increased substantially from 486 in 2000 to 6,248 in 2017 (*3*). Despite frequent exposure to SFGR, gaps remain in our understanding of pathogenic *Rickettsia* spp., disease severity, and tick vectors.

In the United States, several SFGR species, including *R. parkeri*, *R. rickettsii*, and *R. rickettsii* subsp. *californica*, cause disease in humans (*4*,*5*). Among those species, *R. rickettsii* is the most virulent in dogs and humans and can be fatal without early antibiotic intervention (*6*). In addition to *R. rickettsii*, other SFGR species have been detected in dogs in the United States, including *R. montanensis*, *R. amblyommatis*, and *R. parkeri*, all of which caused asymptomatic infection (*7*,*8*). Until recently, *R. rickettsii* was the only SFGR known to cause disease in dogs in North America. Dogs with RMSF can demonstrate fever, lethargy, neurologic signs, and generalized or localized pain, like arthropathy (*9*,*10*). Clinical signs reported in dogs infected with *Rickettsia* sp. 2019-CO-FNY resembled those seen in RMSF, indicating the existence of additional virulent SFGR in the United States and underscoring the importance of expanded vectorborne disease surveillance for canine and human health.

In this study, we cultured and sequenced a novel, pathogenic SFGR, *Rickettsia* sp. 2019-CO-FNY. We identified *Rickettsia* sp. 2019-CO-FNY in 14 additional sick dogs and cultured it from 1 infected dog. On the basis of whole-genome sequencing (WGS) and imaging, we determined that *Rickettsia* sp. 2019-CO-FNY is a new *Rickettsia* species, which we propose naming *Rickettsia finnyi* sp. nov., strain 2024-CO-Wats.

## Methods

### Infected Dogs

All dogs naturally infected with *Rickettsia* sp. 2024-CO-Wats were identified after samples were submitted to a veterinary diagnostic laboratory for canine comprehensive vectorborne disease testing. Signalment, sample collection date, and geographic location were included on submission forms. Attending veterinarians were asked to provide historical and clinical information. Ethical approval for animal use was not required for blood samples initially submitted for diagnostic testing; however, additional blood samples requested for *R. rickettsii* indirect immunofluorescence assay (IFA), posttreatment quantitative PCR (qPCR), and culture were approved under Institutional Animal Care and Use Committee protocol number 21–274. Analysis combined newly acquired data from 14 dogs with data from 3 dogs previously described (*1*).

### *Rickettsia* Detection and Culture

The EDTA whole-blood sample used for culture was collected from a dog on April 25, 2024, by a veterinarian in Indiana, USA, and submitted to a veterinary diagnostic laboratory for comprehensive tickborne disease testing. The sample was received April 30, 2024, and stored at 4°C for 72 hours before testing. Tests consisted of qPCR for vertebrate GAPDH (internal control), *Anaplasma*, Apicomplexa, *Babesia*, *Bartonella*, *Ehrlichia*, hemotropic *Mycoplasma* and *Rickettsia*; IFA for *Babesia vogeli*, *Bartonella henselae*, *Bartonella koehlerae*, *Bartonella vinsonii* subsp. *berkhoffii*, *Ehrlichia canis*, and *Rickettsii* spp.; ELISA for antibodies to *Babesia gibsoni* and a SNAP 4DX Plus point-of-care ELISA (Idexx Laboratories, https://www.idexx.com) for *Dirofilaria immitis* antigen and species-specific antibodies to *E. canis*, *Ehrlichia ewingii*, *Anaplasma phagocytophilum*, *A. platys*, and *Borrelia burgdorferi* (*11*–*17*). *Rickettsia* sp. 2024-CO-Wats infection was confirmed with amplicon sequencing (GENEWIZ, http://www.genewiz.com) of the *Rickettsia* 23s-5s internal transcribed spacer genus qPCR and a newly developed *R. finnyi* species-specific (sp-sp) hydrolysis probe-based qPCR (*1*) (Appendix).

We added blood from a dog naturally infected with *Rickettsia* sp. 2024-CO-Wats to continuously maintained cell cultures using a previously published protocol (*18*). In brief, we combined 100 µL of blood and sucrose-phosphate-glutamate in a 1:1 ratio for each inoculation of 5 replicate cultures of Vero E6 (VE6) and 3 replicate cultures of DH82 and 030D cells seeded in either 7 ml tissue culture tubes, 6-well plates, or T-25 flasks (Fisher Scientific, http://www.fishersci.com) (Appendix Table 1). Cultures were grown at 34°C with 5% CO_2_ in either DMEM 5% FBS (VE6) or RPMI 1640 GlutaMAX 10% FBS (030D and DH82 cells) in a Biosafety Level 3 laboratory. We tested culture supernatants or cell suspensions from passages by qPCR and calculated fold changes in *Rickettsia* (Appendix). We performed retrospective qPCR and amplicon sequencing on stored culture DNA samples to assess a mutation acquired in a major facilitator superfamily (MFS) transporter gene (Appendix) (Table 1). We stained culture samples using the Gimenez method (*19*). We obtained images under oil immersion with an Olympus BX60 microscope and digital camera. We developed and performed an immunofluorescence technique on all 3 infected cell lines and acquired images with BZ-X810 Keyence (Appendix).

**Table 1:** Growth of *Rickettsia* *finnyi* sp. nov. strain 2024-CO-Wats in Vero E6, 030D, and DH82 cells monitored through changing Cq values in *Rickettsia* 23s-5s ITS and *R. finnyi*–specific M61 qPCRs in study of isolation and characterization of novel pathogenic spotted fever group *Rickettsia* in dogs, United States*

Day	Vero E6				030D				DH82		
	Sample	*Rickettsia* 23S-5S (Cq)	*R. finnyi* M61 probe (Cq)		Sample	*Rickettsia* 23S-5S (Cq)	*R. finnyi* M61 probe (Cq)		Sample	*Rickettsia* 23S-5S (Cq)	*R. finnyi* M61 probe (Cq)
3	VE6–5†	28.88	NA		030D-3†	33.32	NA		DH82–3†	31.35	NA
6	VE6–5†	28.88	NA		030D-3-P1†	35.13	NA		DH82–3-P1†	33.38	NA
10	VE6–5†	28.41	32.26		030D-3-P1†	30.37	34.52		DH82–3-P1†	32.83	35.76
13	VE6–5-P1†	30.40	34.28		030D-3 -P1†	30.86	34.45		DH82–3-P1†	28.93	32.60
20	VE6–5-P2†	21.41	25.88		030D-3-P2†	25.27	29.48		DH82–3-P2†	24.74	29.31
32	VE6–5-P4†‡	16.06	17.20		030D-3-P4†	20.35	21.67		DH82–3-P4†	19.49	22.78
57	NA	NA	NA		NA	NA	NA		DH82–3-P7†	18.70	NA
89	VE6–5-P7	12.81	NA		NA	NA	NA		NA	NA	NA
104	VE6–5-P9†‡	15.73	NA		030D-3-P15†	18.59	NA		DH82–3-P14†	18.93	NA

*Cq, quantification cycle; NA, not applicable; qPCR, quantitative PCR. †qPCR performed to identify an MFS transporter single nucleotide mutation T→G. ‡MFS transporter single nucleotide mutation A→C present

### DNA Extraction and Whole-Genome Sequencing

We grew canine 030D and monkey VE6 cells infected with *Rickettsia* sp. 2024-CO-Wats in T25 flasks for DNA extraction (QIAGEN, https://www.qiagen.com). Sequencing was performed by the University of Delaware DNA Sequencing and Genotyping Center using Pacific Biosciences (https://www.pacb.com) Single-Molecule DNA for each culture DNA was sheared to 15 kb using the Megarupture 3 instrument (Diagenode, https://www.diagenode.com). SMRTbell DNA libraries were constructed according to the PacBio HiFi SMRTbell protocol using SMRTbell Express Template Prep Kit 3.0 and barcoded with the SMRTbell adaptor index plate 96A (Pacific Biosciences). AMPure PB beads were diluted at a 3.1× ratio to remove fragments <5 kb before sequencing and 1 SMRTcell was used to sequence the libraries on the Revio PacBio instrument for 30 hours.

### Genome Assembly and Annotation

We assessed sequencing generated by PacBio HiFi circular consensus sequencing reads for quality using NanoPlot (*20*), aligned with host cell DNA, *Canis lupus familiaris* (030D) genomic DNA (Genbank no. GCA_000002285.4), and *Chlorocebus sabaeus* strain WHO RCB 10–87 (VE6) genomic DNA (Genbank no. GCA_015252025.1) using Minimap2 version 2.28 (*21*). We then filtered using SAMtools version 1.20 (*22*) and confirmed host DNA removal and coverage of the *Rickettsia* genome by using BAM file statistics. We assembled unmapped reads using Flye version 2.9.5 (*23*) and assessed quality using version 5.3 of the QUAST tool (*24*). We verified genome completeness and absence of plasmid sequences using Benchmarking Universal Single-Copy Orthologs tool version 5.8.0 and SourceFinder version 1.0 (*25*). We annotated the genome with the National Center for Biotechnology Information Prokaryotic Genome Annotation Pipeline.

### Genomic and Phylogenetic Analysis

We directly compared the whole genomes of 37 *Rickettsia* spp. from GenBank, including 4 strains of *R. rickettsii* and 4 strains of *R. parkeri*, with the genome of 2024-CO-Wats (Appendix Table 2). We analyzed digital DNA-DNA hybridization (dDDH) with the Type Strain Genome Server (https://tygs.dsmz.de) (*26*) and determined average nucleotide identity (ANI) using the OrthoANI tool (*27*). We annotated 38 *Rickettsia* genomes with Prokka version 1.14.6 (default settings, *Rickettsia*-specific BLAST DB) (*28*). We identified orthologous core genes present in all genomes were identified using ProteinOrtho version 6.3.1 (*29*), aligned each gene at the nucleotide level with MAFFT version 7.526 (*30*), and concatenated into a matrix. We then performed maximum-likelihood phylogenetic inference with RAxML-NG version 1.2.1 (*31*) under a general time-reversible plus FC plus gamma 4m plus B model with per-partition parameter estimation. We mapped bootstrap support (500 replicates) onto the best maximum-likelihood tree, which was rooted with *Rickettsia bellii*.

## Results

### Infected Dogs

**Figure 1:**
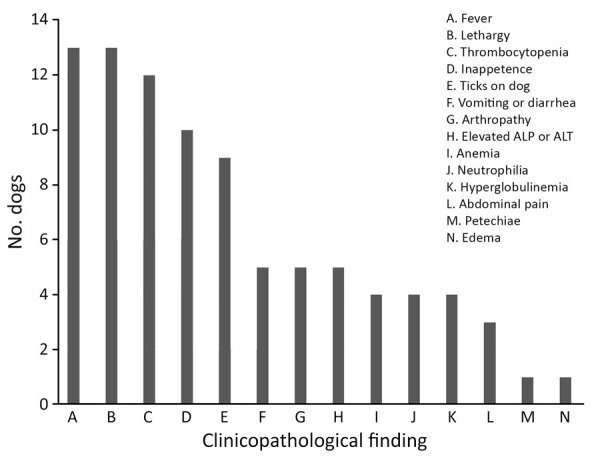
Common clinicopathological findings in 17 dogs naturally infected with *Rickettsia* *finnyi* sp. nov. strain 2024-CO-Wats in study of isolation and characterization of novel pathogenic spotted fever group *Rickettsia* in dogs, United States. ALP, alkaline phosphatase; ALT, alanine transaminase.

We compiled signalment, collection date and location, and vectorborne diagnostic results from the 17 *Rickettsia* sp. 2024-CO-Wats infected dogs (14 new and 3 previously described) (Table 2) (*1*). More than half the samples were collected in May (9/17, 53%), and many dogs (7/17, 41%) resided in Kansas, Missouri, or Oklahoma (Table 2). Two dogs were co-infected with *Babesia* sp. (Coco) or *Mycoplasma hematoparvum*, and 5 dogs were seroreactive for other vectorborne pathogens. Because of limited and inconsistent clinical data, dogs were not uniformly evaluated for each parameter. The most common abnormal findings were fever (n = 13), lethargy (n = 13), and thrombocytopenia (n = 12) (Figure 1). Fourteen veterinarians administered doxycycline therapy (5–10 mg/kg every 12 hours) at the time of sample submission. One dog died before diagnosis and doxycycline therapy, and 1 was euthanized 1 day after starting doxycycline. One dog died because of nephrotic syndrome after treatment as previously described (*1*). Seven veterinarians sent additional samples for *Rickettsia* qPCR testing and *R. rickettsii* IFA. Results for all dogs tested for *Rickettsia* after doxycycline treatment were negative by qPCR, and all but 1 dog had a 4-fold or greater increase in *R. rickettsii* IFA titers (Table 2).

**Table 2:** Date, location, signalment, and vectorborne disease diagnostic results from dogs naturally infected with *Rickettsia* *finnyi* sp. nov. strain 2024-CO-Wats in study of isolation and characterization of novel pathogenic spotted fever group *Rickettsia* in dogs, United States*

Dog no.	Date	State	Breed	Age, y/sex	*Rickettsia* 23S-5S ITS PCR (Cq, Mt)†	*R. rickettsii* IFA titer	Vectorborne disease co-infections, test type (titer)
1‡	2018 May 17	TN	MB	10/MC	37.7, 77.5°	1:512	None
2‡	2019 May 8	IL	BT	9/MC	39.1, 78°	1:256	None
	2019 May 15§				ND	1:8,192	
3‡	2019 Aug 28	OK	MB	9/MC	33.8, 77.5°	1:1,024	None
	2019 Sep 10§				ND	1:8,192	
4	2020 May 3	OK	V	8/MC	38.1, 78°	1:256	*Ehrlichia* SNAP4DxPlus
	2020 May 28§				ND	1:512	
5	2020 May 7	TX	MB	7/MC	34.5, 77°	1:512	*Mycoplasma hematoparvum* PCR+
6	2020 Jul 16	NC	KSD	2/FS	35.4, 77.5°	<1:32	*Bartonella henselae* IFA (1:64)
	2020 Aug 19§				ND	1:1,024	
7	2020 Aug 1	CO	ESS	12/FS	33.8, 77.5°	<1:32	*Babesia* sp. (Coco) PCR+
8	2022 May 23	KS	LR	4/MC	37.7, 77.5°	1:265	*Ehrlichia* SNAP4DxPlus
9	2022 May 24	MO	LR	6/M	38.3, 77.5°	1:2,048	None
	2022 Jun 17§				ND	1:8,192	
10	2023 May 5	KS	ASP	3/FS	33.9, 77.5°	<1:32	None
11	2023 May 5	KS	MB	13/MC	36.5, 77.5°	1:4,096	*Anaplasma* SNAP4DxPlus, *B. canis* IFA (1:256),
12	2023 Sep 13	AL	MB	8/MC	36.6, 78°	1:512	None
	2023 Oct 2§				ND	1:4,096	
13	2023 Sep 18	MO	LR	8/M	33.1, 77.5°	1:256	None
14	2023 Oct 10	IA	GR	5/FS	37.4, 78°	1:64	*Ehrlichia* SNAP4DxPlus, *E. canis* IFA (1:64)
	2023 Oct 19§				ND	1:4,096	
15¶	2024 Apr 25	IN	MB	9/M	28.8, 77°	1:1,024	None
16	2024 Oct 23	LA	TP	4/M	36.5, 77.5°	1:2,048	None
17	2025 May 14	LA	MB	8/MC	36.4, 77.5°	1:4,096	None

*ASP, American Staffordshire terrier; BT, Boston terrier; Cq, quantification cycle; ESS, English springer spaniel; FS, female spayed; GR, golden retriever; IFAT, indirect immunofluorescent antibody; ITS, internal transcribed spacer; KSD, Kangal shepherd dog; LR, labrador retriever; MC, male castrated; Mt, melting temperature; MB, mixed breed; ND, not detected; TP, toy poodle; VBD, vectorborne disease; V, Vizsla. †Rickettsia 23S-5S ITS amplicons were Sanger sequenced and 100% identical to Rickettsia sp. strain 2019-CO-FNY. ‡Dogs described previously (*1*). §Post doxycycline treatment. ¶Sample used for culturing.

### *Rickettsia* Culture and Visualization

**Figure 2:**
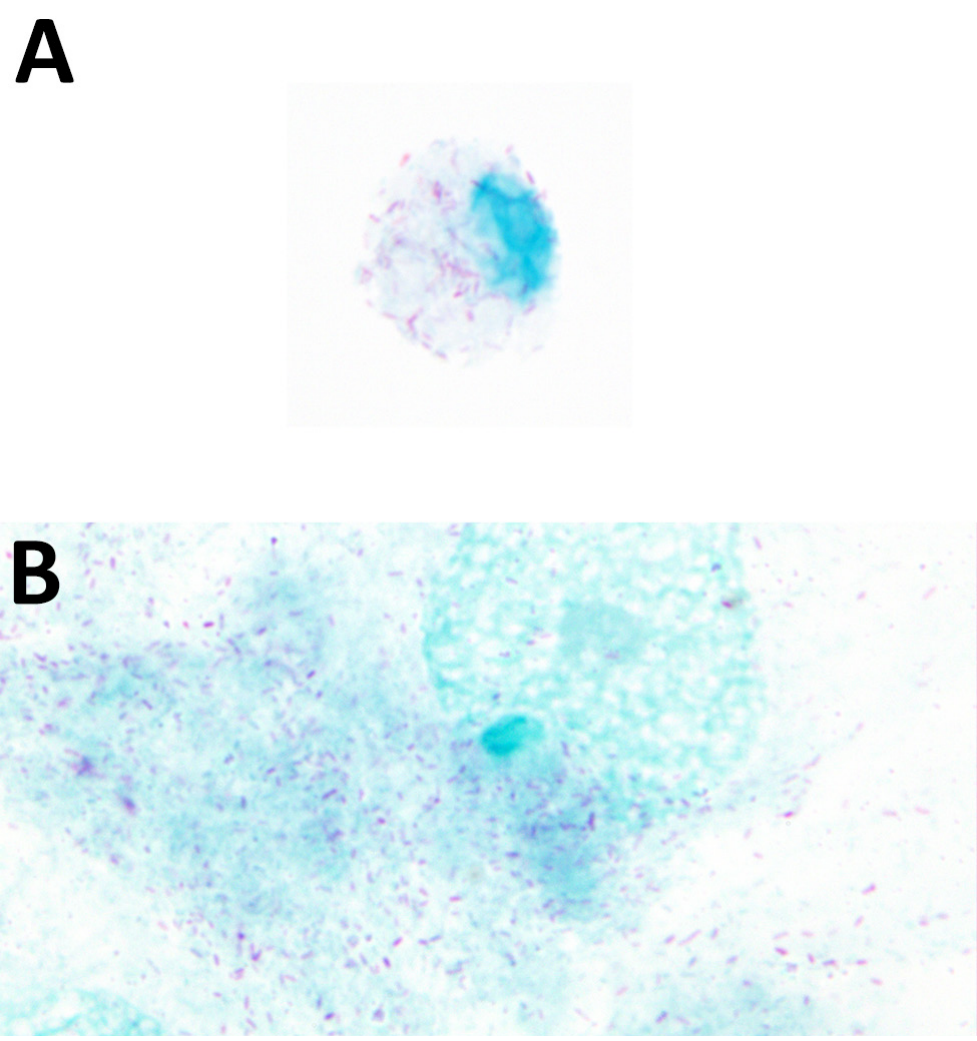
Microscopic images from study of isolation and characterization of *Rickettsia finnyi*, novel pathogenic spotted fever group *Rickettsia* in dogs, United States. Images depict *Rickettsia finnyi* sp. nov. strain 2024-CO-Wats–infected 030D canine mononuclear cells (A) and Vero E6 primate epithelial cells (B) stained with Giménez. Original magnification ×100. Image is white balance adjusted.

**Figure 3:**
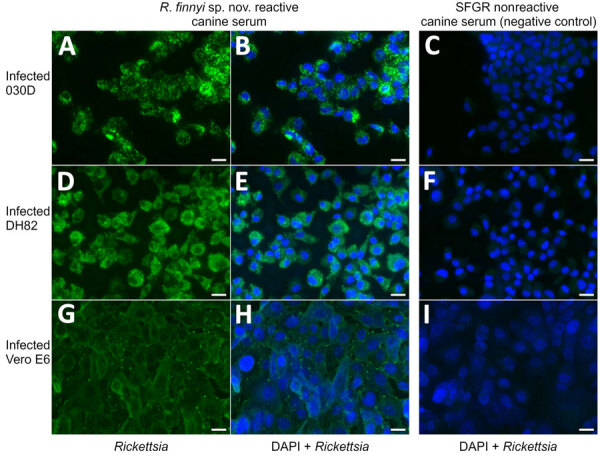
Microscopic images from study of isolation and characterization of *Rickettsia finnyi*, novel pathogenic spotted fever group *Rickettsia* in dogs, United States. Images depict *R.* *finnyi* sp. nov. strain 2024-CO-Wats–infected 030D canine mononuclear cells (A–C), DH82 canine histiocytic cells (D–F), and Vero E6 primate epithelial cells (G–I) detected by immunofluorescence using *R. finnyi* sp. nov.–seroreactive canine serum (dog 2, May 15 date from Table 2). Scale bar indicates 20 µm. SFGR nonreactive canine serum was used as a negative control. Green represents 2024-CO-Wats organisms. Blue represents nuclei of individual mammalian host cells (DAPI). SFGR, spotted fever group *Rickettsia*.

The EDTA-whole blood sample used for culturing was qPCR-positive for *Rickettsia* by 23s-5s ITS qPCR (quantification cycle 28.7, melting temperature 77°C) and *R. finnyi*–specific probe-based qPCR (quantification cycle 31) (Table 2). Serum from the same dog was seroreactive by *R. rickettsii* IFA with a titer of 1:2,048. All other vectorborne tests were negative (Table 2). All 3 cell lines were infected with *Rickettsia* sp. 2024-CO-Wats and maintained over multiple passages (Table 1; Appendix Figure 2). Images of 2024-CO-Wats-infected 030D-P18 and VE6-P4 cells stained with Gimenez revealed red, small (<0.5 by 2 µm), intracytoplasmic, randomly distributed rod-shaped bacteria (Figure 2). We visualized bacteria by immunofluorescence staining in 3 infected cell lines using serum from 2 dogs naturally infected with *Rickettsia* sp. 2024-CO-Wats (Figure 3). The staining did not differ between the 2 serum samples. The 030D-P9 (100% of cells), DH82-P8 (≈95%) and VE6-P2 (100%) cells were highly infected. The bacteria were in the cytoplasm as single rods or, less frequently, as clusters. In addition, we visualized aggregates of bacteria and, to a lesser extent, single bacilli on the cytoplasmic membrane of VE6 cells. We did not observe bacteria with nonreactive serum or with secondary antibody control.

### Whole-Genome Sequencing, Assembly, and Annotation

We performed whole-genome sequencing using 1.33 µg (purity 1.92 A260/A280) of DNA from 030D cells infected with *Rickettsia* sp. 2024-CO-Wats and using 1.73 µg (purity 1.84 A260/A280) of DNA from VE6 cells infected with *Rickettsia* sp. 2024-CO-Wats. PacBio sequencing generated ≈1.5 million reads from infected 030D cells, where 1,481,022 reads were mapped to the host genome and removed leaving 16,333 unmapped reads. Approximately 4.6 million reads were generated from infected VE6 cells, where 3,030,649 reads were mapped to the host genome and removed, leaving 1,619,279 unmapped reads. We subsampled reads from VE6 cells and used 24,476 (2%) of the best quality reads for assembly. Flye generated a single, circularized contig of 1,270,764-bp with 32.3% G+C content for 2024-CO-Wats from both cultures. Mean genome coverage for 2024-CO-Wats was 174× from 030D and 261× from VE6 cultures. The assembled genomes showed 100% Benchmarking Universal Single-Copy Orthologs scores (genome completeness) using the Rickettsiales lineage. SourceFinder did not identify sequences originating from plasmids. Both genomes were deposited in Genbank as *Rickettsia* sp. 2024-CO-Wats cultured from 030D cells (accession no. CP170741) and *Rickettsia* sp. 2024-CO-Wats-2 from VE6 cells (accession no. CP187160). Both genomes were identical except a single nonsynonymous mutation at coordinate 646,406-bp (A/C) coding the 62nd amino acid in an MFS transporter protein (GenBank accession nos. XIA57199 and XRJ55031) changing the TTT (phenylalanine) in the 030D culture to TTG (leucine) in the VE6 culture. PCR and amplicon sequencing of the MFS transporter gene from cultures confirmed the mutation only occurred in VE6-P4 cells beginning on day 32 and was still present in VE6-P9 cells on day 104 (Table 1). The National Center for Biotechnology Information Prokaryotic Genome Annotation Pipeline identified 1,425 genes, 1,234 gene open-reading frames, 33 tRNAs, 3 rRNAs, and 4 ncRNAs.

### Genomic and Phylogenetic Analysis

**Figure 4:**
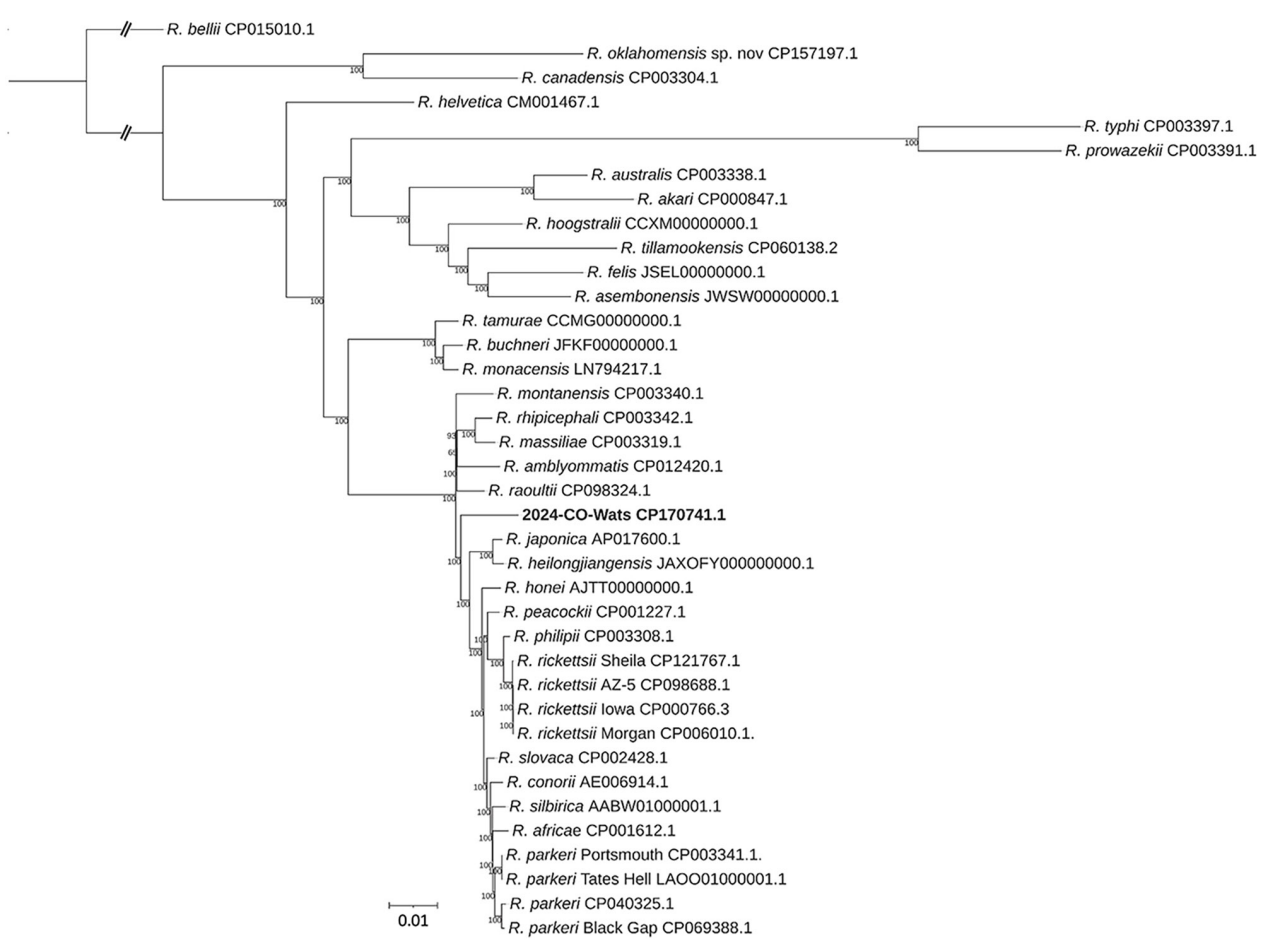
Phylogenetic tree from study of isolation and characterization of *Rickettsia finnyi*, novel pathogenic spotted fever group *Rickettsia* in dogs, United States. Tree depicts 38 *Rickettsia* spp., including *R.* *finnyi* sp. nov. strain 2024-CO-Wats (bold), which has its own distinct branch within the spotted fever group *Rickettsia*. A total of 636 orthologous core genes present in all genomes were aligned at the nucleotide level with MAFFT version 7.526 (*30*) and concatenated into a matrix. Maximum-likelihood phylogenetic inference was performed with RAxML-NG version 1.2.1 (*31*) under a general time-reversible plus FC plus gamma 4m plus B model with per-partition parameter estimation. Bootstrap support (500 replicates) was mapped onto the best maximum-likelihood tree, which was rooted with *R. bellii.*

When compared with 37 *Rickettsia* spp. genomes, the 2024-CO-Wats genome was most similar to *R. raoultii* (GenBank accession no. CP098324); average nucleotide identity was 96.86% and dDDH was 70.6% (Appendix Table 2). ProteinOrtho identified 636 core orthologous genes, and phylogenetic analysis placed 2024-CO-Wats with its own distinct branch within the SFGR (Figure 4).

## Discussion

We isolated and sequenced the genome of a novel, pathogenic SFGR (formerly *Rickettsia* sp. 2019-CO-FNY) from a clinically ill dog that we propose naming *Rickettsia finnyi* sp. nov. type strain 2024-CO-Wats. This novel *Rickettsia* was cultured and maintained over many passages proving viability in epithelial (VE6) and mononuclear (030D and DH82) cells. Whole-genome sequencing generated a small, circular genome (1.27 kb) from infected 030D and VE6 cell lines. The *Rickettsia* genome was identical except for 1 nucleotide mutation in the VE6 cultured strain in an MFS transporter protein gene. The difference resulted in a conservative hydrophobic-to-hydrophobic amino acid change (phenylalanine to leucine), which could affect substrate specificity and transport efficiency, among other functions. The mutation could possibly have occurred in the VE6 culture because of different growth conditions or passage techniques. Genome alignment revealed it was most similar to *R. raoultii* (CP098324), with relatively small percentage differences between other SFGR species. Phylogenetic analysis of the genome sequence (CP170741) placed *R. finnyi* sp. nov. (2024-CO-Wats) on a distinct lineage within the spotted fever group, further supporting that it is a new species.

Criteria to designate a new *Rickettsia* sp. indicate that the genome must have an OrthoANI value of >83.63% compared with >1 *Rickettsia* species with a validly published name to be classified in the genus and a dDDH value of <92.3%, OrthoANI value of <99.19% identical with other known *Rickettsia* spp., or both to be considered a new species (*32*). *R. finnyi* sp. nov. (2024-CO-Wats) meets each of those criteria to be recognized as a new *Rickettsia* species. Genome comparisons revealed the highest results from both metrics was *R. raoultii* (CP098324) with a dDDH formula 2 value of 70.6% and OrthoANI measurement of 96.86%.

By both Gimenez staining and immunofluorescence, the morphology and cellular localization of *R. finnyi* sp. nov. (2024-CO-Wats) revealed characteristics consistent with pathogenic SFGR, including high quantities of bacillary-shaped intracellular bacteria and evidence of cell-to-cell expansion. In epithelial cells, *R. finnyi* sp. nov. (2024-CO-Wats) concentrated at the cytoplasmic membrane, likely representing direct transfer to neighboring cells, a well-documented mechanism used by SFGR for intracellular expansion (*33*). In contrast, the cytoplasmic localization observed in mononuclear cells is typical of obligate intracellular rickettsiae and provides supporting evidence that *R. finnyi* sp. nov. (2024-CO-Wats) is well adapted to survive in nonendothelial mammalian host cells (*34*).

In this study, we did not assess in vitro pathogenicity or cytopathic effects of *R. finnyi* sp. nov. (2024-CO-Wats); however, we documented growth for >104 days in 2 mononuclear cell lines. Previous studies have reported that pathogenic *Rickettsia* spp. can proliferate in nonendothelial cells, including leukocytes and macrophages, and that they exhibit enhanced intracellular survival in macrophage-like cells; the nonpathogenic *Rickettsia* spp. lacked this ability (*34*–*38*). *Rickettsia* spp. capable of proliferating in phagocytic cells have likely adapted mechanisms to evade host immunity and replicate before invading endothelial cells. The prolonged survival of *R. finnyi* sp. nov. (2024-CO-Wats) in mononuclear cells, along with clinical signs observed in naturally infected dogs, provide evidence that it is pathogenic. Additional studies comparing transcription levels and posttranslational modifications of *R. finnyi* sp. nov. in phagocytic versus epithelial cells might help elucidate mechanisms of pathogenicity and cytologic variation.

Since our initial description in 2020 of 3 clinically ill dogs naturally infected with *R. finnyi* sp. nov., an additional 14 infected dogs have been identified. Historical and clinicopathological findings for most of the dogs included a combination of fever, lethargy, and thrombocytopenia, like those seen in *R. rickettsii* infections. Vectorborne co-infections could have contributed to the abnormalities or disease severity in those dogs. However, the presence of similar abnormalities seen in the other dogs in our study without evidence of co-infections further supports the notion that *R. finnyi* sp. nov. is pathogenic in dogs.

Several *Rickettsia* spp., including *R. parkeri* and *R. rickettsii*, have been documented in both dogs and humans (*6*,*8*,*39*). Dogs serve as sentinels for human rickettsiosis, particularly RMSF, because they share similar clinical signs and exposure to the same ticks that transmit *R. rickettsii* (*6*,*39*,*40*). Antibodies to *R. finnyi* sp. nov. cross-react with *R. rickettsii* in IFA, as with most SFGR, making it challenging to accurately diagnose RMSF or other spotted fever rickettsiosises in dogs and humans. Furthermore, diagnostic PCRs specific to *R. rickettsii* might not detect novel *Rickettsia* spp. For example, *Rickettsia* sp. CA6269, which represents a novel *Rickettsia* sp. or subspecies of *R. rickettsii*, was detected in humans using broad-based *Rickettsia* qPCR screening after negative *R. rickettsii* and *R. typhi* sp-sp qPCR results (*41*). Studies are needed to determine whether *R. finnyi* sp. nov. can also infect and cause disease in humans.

*R. finnyi* sp. nov. (2024-CO-Wats) is likely transmitted by the lone star tick, *Amblyomma americanum*. Indeed, Noden et al. (*42*) reported amplified DNA sequences that were 100% identical with *Rickettsia* sp. 2019-CO-FNY in an *A. americanum* tick collected in 2018 in Oklahoma. Supporting possible exposure to *A. americanum* ticks, 1 *R. finnyi* sp. nov.–infected dog was co-infected with *Babesia* sp. coco, a protozoan pathogen detected in *A. americanum* ticks (*43*,*44*). Moreover, the geographic range of *A. americanum* ticks overlaps with areas in the United States where most infected dogs have been identified to date. Given the zoonotic potential of many *Rickettsia* spp., identifying the vectors and reservoir hosts of *R. finnyi* sp. nov. is essential toward understanding its transmission dynamics and potential public health impacts.

In conclusion, *Rickettsia finnyi* sp. nov. (fin′ny.i. N.L. gen. n. *finnyi*, named after Finny, the first infected dog, in recognition of companion dogs that have contributed to the discovery of novel pathogens) is proposed as a novel spotted fever group *Rickettsia*. Cells are small (<0.5 µm by 2 µm), rod-shaped intracytoplasmic bacteria that stain red using the Gimenez technique and grow in epithelial (Vero E6) and mononuclear (DH82 and 030D) cell lines. The circular genome is 1.27 Mb. This species has been identified in *A. americanum* ticks from Oklahoma and in dogs from the central and southeastern United States, where infection was associated with moderate to severe illness. The type strain is 2024-CO-Wats, isolated from a naturally infected dog in Tippecanoe County, Indiana, in 2024. Cultures have been deposited in 2 curated rickettsial banks: the Centers for Disease Control and Prevention Rickettsial Isolate Reference Collection (WDCM 1093; accession no. RFI001), Atlanta, Georgia, USA; and the Collection de Souches de l’Unité des Rickettsies (WDCM 875; accession no. R5053), Marseille, France.

Appendix Additional information about the isolation and characterization of *Rickettsia finnyi*, novel pathogenic spotted fever group *Rickettsia* in dogs, United States
