# Supplementary material for: Isolation and Characterization of Rickettsia finnyi, Novel Pathogenic Spotted Fever Group Rickettsia in Dogs, United States
Source: Emerg Infect Dis. 2025 Nov;31(11):2118–27. doi: 10.3201/eid3111.250681 (PMC12704542; doi:10.3201/eid3111.250681)
Supplement: Appendix — Additional information about the isolation and characterization of Rickettsia finnyi, novel pathogenic spotted fever group Rickettsia in dogs, United States [file 25-0681-Techapp-s1.pdf]

Article DOI: https://doi.org/10.3201/eid3111.250681

*EID cannot ensure accessibility for supplementary materials supplied by authors. Readers who have difficulty accessing supplementary content should contact the authors for assistance.*

# Isolation and Characterization of *Rickettsia finnyi*, Novel Pathogenic Spotted Fever Group *Rickettsia* in Dogs, United States

## Appendix

### *Rickettsia finnyi* sp. nov strain 2024-CO-Wats M61 glycyl aminopeptidase qPCR

The *R. finnyi* sp-sp qPCR primers (5′CTTAGAAATGGGGACATA GTTAATG 3′ and 5′GTATATATCTTTTTTATTAGCACTTG 3′) and probe (6-FAM/ZEN- ATCAGATAG TTACAATCAACACAATTAAAGGC-IBFQ) were created (Integrated DNA Technologies, http://www.idtdna.com) to amplify a 147-bp region of the M61 glycyl aminopeptidase gene based on sequence alignment (Geneious Prime version 2021.1.1, https://www.geneious.com) with *Rickettsia* sp. 2019-CO-FNY M61 glycyl aminopeptidase gene region (OQ383420) and multiple *Rickettsia* spp. sequences available in NCBI GenBank (Appendix Figure 1). Amplification assays were performed in CFX96 Real-Time Detection System combined with C1000 Thermal Cycler (Bio-Rad, USA). *Rickettsia finnyi* sp-sp qPCRs contained 12.5 µL Primetime Gene Expression Master Mix (Integrated DNA Technologies, http://www.idtdna.com), primers at 0.6 µM and probe at 0.4 µM, 5 µL of DNA template and molecular-grade water to a final volume of 25 µL. Thermocycler conditions consisted of an initial denaturation step at 98°C for 3 minutes, followed by 40 cycles of 98°C for 15 s and 60°C for 30 s.

### Major facilitator superfamily (MFS) transporter qPCR

Primers (5′ CAAGCAGTCGGATTACTTTC 3′ and 5′ AAACAACTACAATCTTACGTCC 3′) were designed to amplify a major facilitator

superfamily (MFS) transporter gene region for retrospective assessment of a mutation acquired in culture. Amplification assays were performed in CFX96 Real-Time Detection System combined with C1000 Thermal Cycler (Bio-Rad, USA). *Rickettsia finnyi* sp-sp qPCRs contained 12.5 µL Primetime Gene Expression Master Mix (Integrated DNA Technologies, http://www.idtdna.com), primers at 0.4 µM, 5 µL of DNA template and molecular-grade water to a final volume of 25 µL. Thermocycler conditions consisted of an initial denaturation step at 98°C for 3 minutes, followed by 40 cycles of 98°C for 15 s, 58°C for 15 s and 72°C for 15 s.

All PCR assays were run with negative molecular-grade water, a negative control of known uninfected canine DNA, and a positive control.

## *Rickettsia* sp. 2024-CO-Wats Cultures.

Cell cultures 030D, DH82 and Vero E3 grown in varying culture containers were infected with *Rickettsia* sp. 2024-CO-Wats and monitored through culture supernatants or cell suspensions collected at various passages (P) (Appendix Tabel 2). DNA was extracted from 100 µL using a DNA extraction kit (Qiagen, https://www.qiagen.com) and monitored by *Rickettsia* 23s-5s ITS and GAPDH qPCRs. Fold changes in *Rickettsia* were calculated as $2^{-\Delta\Delta Cq}$, in which $\Delta\Delta Cq = (Cq_{Rick\ 23–5} – Cq_{GAPDH})$ timex – $(Cq_{Rick\ 23–5} – Cq_{GAPDH})$ time0 (Appendix Figure 2). At P4, one *Rickettsia* sp. 2024-CO-Wats culture from each cell line replicate was selected for continued maintenance, where 030D-P24 and DH82-P22 were maintained for 156 days and VE6-P4 cells for 108 days

## Immunofluorescence staining

Infected cells were seeded on Nunc Lab-Tek II CC2 8-well chamber slides (ThermoFisher, https://www.thermofisher.com) from the following cultures and passages: 030D-P9, DH82-P8, and VE6-P2. At ~90% confluency, cells were rinsed with PBS and treated with 100 µg/ml of gentamicin sulfate for 2.5 h. Cells were washed 2 times to remove extracellular bacteria and residual antibiotics. They were fixed with 10% neutral buffered formalin for 10 min, permeabilized with 0.3% Triton X-100 in PBS at room temperature for 30 min and blocked with goat serum, Tween-20, and 0.5% of powdered nonfat milk for 1 h. Cells were incubated overnight at 4°C with canine serum diluted at 1:500 in a humidified chamber. Serum samples

used for incubation were derived from dogs numbered 2 and 3 infected with *Rickettsia* sp. 2024-CO-Wats that were cross-reactive against *R. rickettsii* IFA at 1:8192 (Table 1). The negative serum control was obtained from an archived, canine sample previously tested by a comprehensive tick-borne disease panel that was PCR negative and serologically nonreactive for all tick-borne pathogens tested, including *R. rickettsii* IFA. Infected cells were incubated with a fluorescein Isothiocyanate (FITC)-conjugated goat anti-dog IgG (H+L) antibody (Sigma-Aldrich, https://www.sigmaaldrich.com) for 1 h at room temperature. A secondary antibody control (blocking solution instead of canine serum) was performed. Nuclei were counterstained using DAPI (ThermoFisher, https://www.thermofisher.com). Between sequential steps (except after the blocking step), cells were washed 3 times with PBS. Coverslips were mounted with Prolong Gold antifade reagent (Invitrogen, https://www.thermofisher.com). Images were acquired with BZ-X810 Keyence.

**Appendix Table 1.** A summary of conditions used for *Rickettsia* sp. 2024-CO-Wats inoculation in Vero E6 (VE6), 030D, and DH82 cell lines and which conditions produced growth.

| Culture* | Container surface area ($cm^2$) | Cell confluency (%) | Blood volume (µL)† | Days between blood collection to inoculation | Media | Media volume (mL) | Incubation (days) | Growth detected‡ |
|---|---|---|---|---|---|---|---|---|
| VE6–1 | 7mL Tube (5.5) | 95 | 100 | 7 | DMEM 5% FBS | 1.5 | 12 | Yes |
| VE6–2 | 7mL Tube (5.5) | 95 | 100 | 7 | DMEM 5% FBS | 1.5 | 12 | Yes |
| VE6–3 | 7mL Tube (5.5) | 50 | 100 | 7 | DMEM 5% FBS | 1.5 | 12 | Yes |
| VE6–4 | 7mL Tube (5.5) | 50 | 100 | 7 | DMEM 5% FBS | 1.5 | 12 | Yes |
| VE6–5 | T25 Flask (25) | 75 | 300 | 8 | DMEM 5% FBS | 4.5 | 11 | Yes |
| 030D-1 | 6-well Plate (9.5) | 75 | 100 | 8 | RPMI 10% FBS | 1.5 | 4 | Yes |
| 030D-2 | 6-well Plate (9.5) | 75 | 100 | 8 | RPMI 10% FBS | 1.5 | 4 | Yes |
| 030D-3 | 6-well Plate (9.5) | 75 | 100 | 8 | RPMI 10% FBS | 1.5 | 4 | Yes |
| DH82–1 | 6-well Plate (9.5) | 75 | 100 | 8 | RPMI 10% FBS | 1.5 | 4 | No |
| DH82–2 | 6-well Plate (9.5) | 75 | 100 | 8 | RPMI 10% FBS | 1.5 | 4 | No |
| DH82–3 | 6-well Plate (9.5) | 75 | 100 | 8 | RPMI 10% FBS | 1.5 | 4 | Yes |

*Numbers after the cell lines represent replicates. DMEM, Dulbecco's minimum essential medium; FBS, fetal bovine serum; RPMI, Roswell Park Memorial Institute-1640 Medium, GlutaMAX supplement; SPG, sucrose-phosphate-glutamate buffer.
†Combined volume of EDTA whole blood and SPG at a 1:1 ratio.
‡Growth was initially determined based on *Rickettsia* 23S-5S ITS and *R. finnyi*-specific M61 qPCR having lower Cq values than the diagnostic blood sample.

**Appendix Table 2.** *Rickettsia* spp. used in genomic comparison with 2024-CO-Wats using OrthoANI and dDDH GGDC.

| Rickettsia genome compared with R. finnyi CP170741.1 | OrthoANI value (%) | dDDH GGDC f1 | dDDH GGDC f2 | dDDH GGDC f3 | Diff. G+C (%) |
|---|---|---|---|---|---|
| *R. conorii raoultii* CP098324.1 | 96.86 | 91.9 | 70.6 | 91.0 | 0.19 |
| *R. montanensis* CP003340.1 | 96.81 | 94.0 | 69.7 | 92.5 | 0.26 |
| *R. slovaca* CP002428.1 | 96.78 | 92.3 | 70.5 | 91.3 | 0.19 |
| *R. peacockii* CP001227.1 | 96.75 | 87.1 | 69.5 | 86.8 | 0.30 |
| *R. honei* AJTT01 | 96.74 | 92.3 | 69.1 | 91.0 | 0.11 |
| *R. japonica* AP017600.1 | 96.68 | 91.6 | 69.5 | 90.5 | 0.01 |
| *R. parkeri* CP040325.1 | 96.66 | 93.0 | 68.6 | 91.5 | 0.14 |
| *R. sibirica* AABW01 | 96.63 | 91.4 | 68.5 | 90.1 | 0.16 |
| *R. conorii* AE006914.1 | 96.60 | 91.9 | 68.7 | 90.5 | 0.13 |
| *R. massiliae* CP003319.1 | 96.55 | 89.9 | 68.0 | 88.8 | 0.29 |
| *R. philipii* CP003308.1 | 96.55 | 93.8 | 67.5 | 91.9 | 0.16 |
| *R. rhipicephali* CP003342.1 | 96.54 | 90.0 | 68.3 | 88.9 | 0.09 |
| *R. parkeri* CP069388.1 | 96.52 | 91.2 | 67.5 | 89.8 | 0.15 |
| *R. parkeri* CP003341.1 | 96.52 | 91.9 | 67.8 | 90.5 | 0.13 |
| *R. parkeri* LAOO01000001.1 | 96.51 | 91.9 | 67.9 | 90.5 | 0.13 |
| *R. africae* CP001612.1 | 96.51 | 91.6 | 67.5 | 90.1 | 0.10 |
| *R. rickettsii* str. ‘Sheila Smith’ CP121767.1 | 96.48 | 92.6 | 66.9 | 90.8 | 0.16 |
| *R. rickettsii* str. AZ-5 CP098688.1 | 96.44 | 92.0 | 66.8 | 90.3 | 0.15 |
| *R. conorii heilongjiangensis* JAXOFY01 | 96.43 | 93.4 | 67.4 | 91.6 | 0.03 |
| *R. rickettsii* str. Morgan CP006010.1 | 96.39 | 92.0 | 66.7 | 90.3 | 0.15 |
| *R. rickettsii* str. Iowa CP000766.3 | 96.39 | 92.1 | 66.7 | 90.3 | 0.14 |
| *R. amblyommatis* CP012420.1 | 96.28 | 87.0 | 65.6 | 85.9 | 0.10 |
| *R. tamurae buchneri* JFKF01 | 94.24 | 55.9 | 54.4 | 56.4 | 0.17 |
| *R. monacensis* LN794217.1 | 94.16 | 64.3 | 53.5 | 63.5 | 0.08 |
| *R. tamurae* CCMG00000000.1 | 93.57 | 69.4 | 52.1 | 67.5 | 0.16 |
| *R. hoogstraalii* CCXM01 | 92.77 | 65.4 | 47.5 | 62.7 | 0.07 |
| *R. helvetica* CM001467.1 | 92.72 | 64.7 | 48.4 | 62.4 | 0.09 |
| *R. asembonensis* JWSW01 | 92.65 | 62.3 | 47.9 | 60.3 | 0.07 |
| *R. tillamookensis* CP060138.2 | 92.28 | 64.2 | 46.1 | 61.3 | 0.09 |
| *R. felis* JSEL01 | 92.11 | 57.2 | 46.3 | 55.5 | 0.13 |
| *R. australis* CP003338.1 | 91.77 | 65.6 | 44.1 | 61.7 | 0.02 |
| *R. akari* CP000847.1 | 91.31 | 64.0 | 42.0 | 59.7 | 0.03 |
| *R. canadensis* CP003304.1 | 88.62 | 51.3 | 36.4 | 47.6 | 1.29 |
| *R. oklahomensis* CP157197.1 | 88.48 | 52.4 | 35.7 | 48.2 | 1.60 |
| *R. prowazekii* CP003391.1 | 87.42 | 43.9 | 33.1 | 40.7 | 3.30 |
| *R. typhi* CP003397.1 | 87.38 | 41.8 | 32.4 | 38.8 | 3.39 |
| *R. bellii* CP015010.1 | 81.66 | 19.4 | 27.4 | 19.4 | 0.69 |

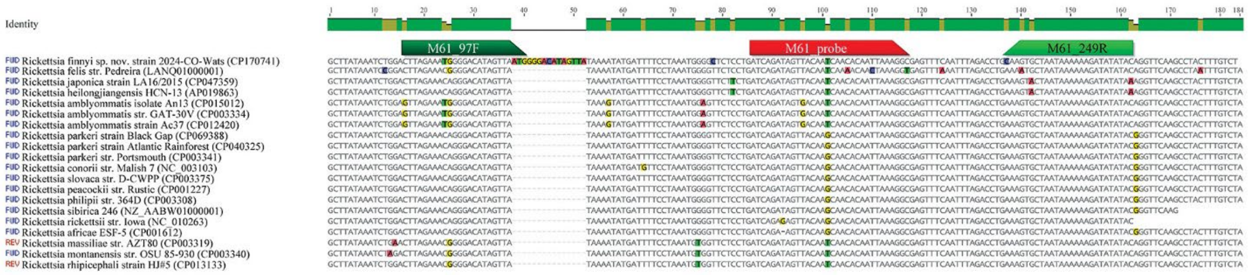


**Appendix Figure 1.** A sequence alignment depicting the oligonucleotides for the *Rickettsia* M61 glycyl aminopeptidase qPCR aligned with *Rickettsia finny* sp. nov. and multiple *Rickettsia* spp. sequences available in NCBI GenBank.

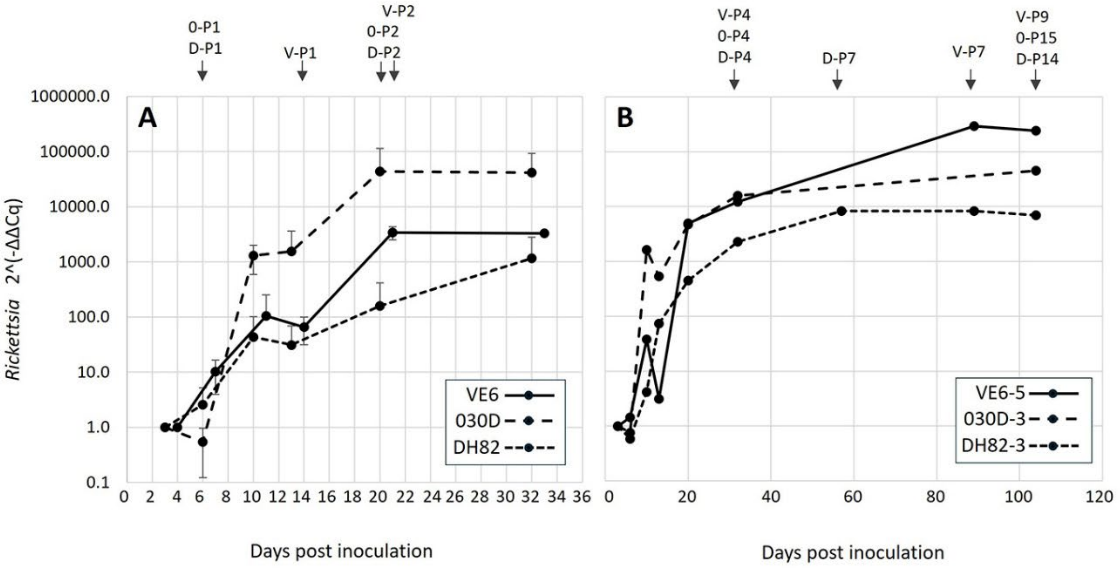


**Appendix Figure 2.** Line graphs depicting the relative growth curve of *Rickettsia finny* sp. nov. 2024-CO-Wats in replicates of Vero E6 (VE6), 030D and DH82 cells measured to 33 days (A) and individual cultures measured to 104 days (B) after inoculation with a naturally infected dog blood sample. *Rickettsia* growth is represented by *Rickettsia* 23s-5S ITs qPCR Cq values normalized to host GAPDH Cq values and fold change (2^(-ΔΔCq) relative to first day of testing after inoculation. Passages are indicated with arrows. 0-P, 030D passages; D-P, DH82 passages; V-P, VE6 passages.
